# Supplementary material for: Identification of photoperiod-induced specific miRNAs in the adrenal glands of Sunite sheep (Ovis aries)
Source: Front Vet Sci. 2022 Jul 22;9:888207. doi: 10.3389/fvets.2022.888207 (PMC9354845; doi:10.3389/fvets.2022.888207)
Supplement: Supplementary file 1 [file Data_Sheet_1.ZIP › Supplementary file/miRDeep2 file.zip!/miRDeep2 file/Novel_Structure/LP42A3/Novel_420.pdf]

Provisional ID : NC_019466.2_11778
Score total : 1.6
Score for star read(s) : -1.3
Score for read counts : 0
Score for mfe : 1.3
Score for randfold : 1.6
Score for cons. seed :
Total read count : 45
Mature read count : 45
Loop read count : 0
Star read count : 0

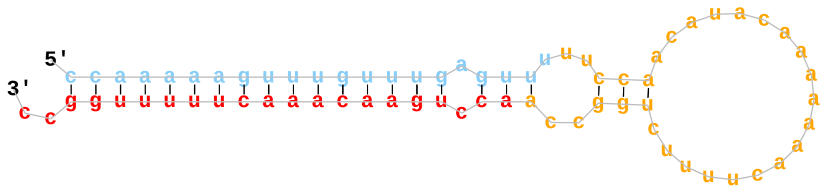

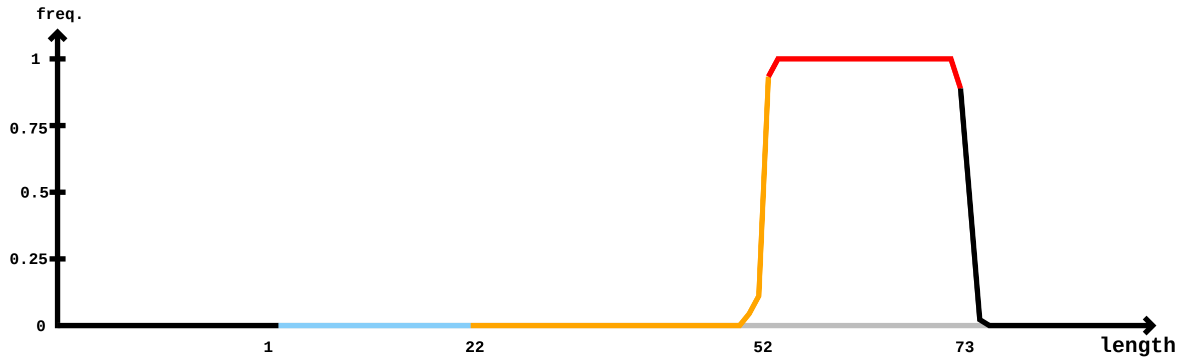

Star Mature

```
5'- ggggugcauguauugaauuggccaaaaaguuuguuugaguuuuuccaacauacaaaaaaacuuuucuggccaaccugaacaaacuuuuuggccaaccccaauuuuuugucca   -3'   exp
    (((((.............(((((((((((((((((((.(((...(((...................)))..))).))))))))))))))))))))))))).............   reads   mm   sample
    ......................................................................caaccugaacaaacuuuuuggc....................   2   0   seq
    .......................................................................aaUcugaacaaacuuuuuggc....................   1   1   seq
    .......................................................................aaUcugaacaaacuuuuuggcc...................   2   1   seq
    ........................................................................aAcugaacaaacuuuuuggc....................   1   1   seq
    ........................................................................aUcugaacaaacuuuuuggcc...................   34   1   seq
    ........................................................................accugaacaaacuuuuuggcc...................   1   0   seq
    ........................................................................aUcugaacaaacuuuuuggccaa.................   1   1   seq
    .........................................................................Ucugaacaaacuuuuuggc....................   1   1   seq
    .........................................................................Ucugaacaaacuuuuuggcca..................   2   1   seq
```
